# Outcome analysis after cephalomedullary nail implantation in older adults and elderly patients with per-, sub- or intertrochanteric femur fractures

**DOI:** 10.1186/s13104-025-07400-2

**Published:** 2025-07-25

**Authors:** Alexander Blümke, Adaugo Okoro, João Pinheiro, Maximilian Mellinghoff, Daniel Kühlwein, Aditya Vadgaonkar, Frederic Bludau, Andreas Schilder, Svetlana Hetjens, Michael Hackl, Sascha Gravius, Ali Darwich

**Affiliations:** 1 Department of Orthopedics and Trauma Surgery, University Medical Center Mannheim, Medical Faculty Mannheim, Heidelberg University, Mannheim, Germany; 2 Department of Medical Statistics and Biomathematics, Medical Faculty Mannheim, Heidelberg University, Mannheim, Germany

**Keywords:** Per-, Sub- and intertrochanteric femur fractures, Cephalomedullary nailing, Elderly patients, Osteoporosis

## Abstract

**Objective:**

Per-, sub-, and intertrochanteric femur fractures are common in older adults and elderly patients. They frequently lead to functional decline and increased dependency. Despite the routine use of cephalomedullary nails (CMN) for stabilization, detailed data on perioperative outcomes in this vulnerable population are scarce.

**Results:**

In this retrospective study, we analyzed 401 patients aged ≥ 50 years who underwent CMN fixation for per-, sub-, or intertrochanteric femur fractures at a university medical center between 2019 and 2024. Pre-, intra-, and postoperative parameters, including demographics, fracture classification, comorbidities, and functional outcomes, were extracted from hospital records and analyzed descriptively. The median patient age was 84 years (IQR 75–89) and 62.6% were female. Most fractures were classified as AO/OTA type A2 (49.9%). Prior to the fracture, 51.6% of patients were fully mobile without aids, yet only 1% maintained this mobility at discharge. The median length of stay was 12 days (IQR 7–18). In-hospital mortality was 8.4%. Postoperatively, hemoglobin and hematocrit levels declined. Discharge destinations included home (36.5%), nursing homes (19.2%), and rehabilitation facilities (12.2%).

## Background

Per-, sub-, and intertrochanteric femur fractures are a significant concern in orthogeriatrics, often leading to profound impacts on mobility and independence. Due to the high risk of complications such as pulmonary embolism, pneumonia, sarcopenia, and pressure ulcers, conservative treatment is seldom recommended. Surgical intervention, particularly cephalomedullary nailing, offers several advantages, including immediate fracture stabilization, which allows for an early full weight-bearing mobilization and reduces the risks associated with prolonged rest and immobilization [1]. Early mobilization is crucial in maintaining neuromuscular function, reducing risk of deep vein thrombosis, and improving overall recovery outcomes [2]. Surgical interventions are associated with several potential complications, including prolonged hospitalization, postoperative infections, non-union, mechanical failure, and persistent postoperative pain, which can adversely impact clinical outcomes [3, 4]. Despite the clinical significance of these fractures, there is limited literature on patient demographics, complication rates, and long-term outcomes, underscoring the need for further research to optimize perioperative care, post-discharge support, and long-term rehabilitation strategies for this vulnerable population [5, 6].

Recent studies have provided valuable insights into the management of per-, sub- and intertrochanteric fractures in elderly patients. A study focusing on patients over 90 years old reported higher in-hospital mortality and complication rates compared to younger cohorts, emphasizing the need for specialized perioperative care in this age group [7]. In addition, surgical intervention in geriatric intensive care unit (ICU) patients resulted in favorable outcomes, suggesting that age alone should not be a contraindication for surgery [8]. Furthermore, research comparing other techniques, such as dynamic hip screws with cephalomedullary nails confirmed that both are effective in treating pertrochanteric fractures, although the choice of implant should be individualized based on fracture type and patient health status [9]. These findings underscore the importance of a comprehensive, and individualized approach to managing pertrochanteric fractures in the elderly, considering patient-specific factors and existing comorbidities to optimize outcomes.

Despite the known benefits of cephalomedullary nailing in elderly patients, existing literature is scarce regarding detailed patient demographics, complication rates, and postoperative outcomes for per-, sub-, and intertrochanteric fractures. This gap limits the development of targeted perioperative and rehabilitation strategies to optimize patient recovery. To address this gap, this retrospective study systematically evaluates patient characteristics, perioperative variables, and functional outcomes following cephalomedullary nail implantation to enhance clinical decision-making and improve patient care in this vulnerable population.

## Methods

This retrospective study analyzed 401 patients who underwent cephalomedullary nailing for a per-, sub-, or intertrochanteric femur fracture at a university medical center over a 5-year period between 2019 and 2024. Since approximately 90% of proximal femur fractures occur in individuals above the age of 50 years, patients younger than 50 years and those with pathological fractures were excluded [10].

Preoperative, intraoperative, and postoperative parameters were systematically assessed and are shown in Table 1. The fracture type was classified according to the AO/OTA classification system [11]. Patient age at surgery, surgical duration, and time from admission to surgery, the difference between preoperative and postoperative hemoglobin values, as well as total hospitalization duration were calculated. Patients' osteoporosis management at admission was assessed. Baseline osteoporosis therapy was defined as supplementation with vitamin D and/or calcium. Specific osteoporosis therapy was defined as pharmacological treatment, including bisphosphonates, denosumab, selective estrogen receptor modulators or osteoanabolically active substance such as romosozumab and teriparatide. Classifications were made according to the current recommendations of the National Osteoporosis Foundation (NOF) and relevant literature [12, 13].

**Table 1:** Overview of evaluated parameters, categorized into preoperative, intraoperative, and postoperative groups

Preoperative parameters
Demographics
Sex
Age at surgery
Date and time of hospital admission
Fracture related
Fracture localization (right / left side)
Fracture type (basicervical, subtrochanteric, pertrochanteric classified acc. to the AO/OTA classification system)
Additional injuries requiring intervention
Pre-fracture mobility status (without assistive devices, walking stick, walker, walking frame, able to stand, wheelchair, bedridden)
Living arrangement (with spouse, with children, with other family, assisted living, short-term nursing care, nursing home, alone)
Comorbidities
Number of pre-existing medical conditions
Number of prescribed medications at admission
American Society of Anesthesiologists (ASA) risk classification score
Charlson Comorbidity Index (CCI)
Body mass index (BMI, kg/m2)
Baseline osteoporosis therapy
Specific osteoporosis therapy
Pre-admission anticoagulant use
Laboratory values
Hemoglobin level at admission (g/dL)
Hematocrit ratio at admission (%)
Leukocyte count at admission (10^9^/L)
Albumin level at admission (g/L)
Intraoperative parameters
Surgical timing
Surgical incision time
Surgical closure time
Surgical duration (in minutes)
Surgical technique
Surgical reduction technique (open or closed)
Application of cerclage wires
Cement augmentation
Implant characteristics
Nail model [Proximal Femoral Nail Antirotation (PFNA), the Trochanteric Femoral Nail Advanced (TFNA) or other]
Anesthesia
Type of anesthesia (general anesthesia or spinal anesthesia)
Intraoperative events
Occurrence of perioperative complications
Postoperative parameters
Functional outcomes
Barthel Index (3.–7. POD)
Rehabilitation
Inclusion of patients in a multidisciplinary complex geriatric rehabilitation program
Complications and interventions
Postoperative blood transfusion
Revision surgery during hospitalization
Intensive care unit (ICU) admission
Discharge information
Date of discharge
Mobility status at discharge (without assistive devices, walking stick, walker, walking frame, able to stand, wheelchair, bedridden)
Post-discharge care setting (home, complex geriatric rehabilitation program within a different dept. of the hospital, rehabilitation facility, short-term nursing care, nursing home, stroke unit, another dept. within the hospital, different hospital, hospice care, death)
Laboratory values
Hemoglobin levels (3.-6. POD, g/dL)
Hematocrit ratio (3.-6. POD, %)
Leukocyte count (3.-6. POD, 10^9^/L)
CRP level (3.-6. POD, mg/L)
Calculated parameters
Difference between preoperative and postoperative hemoglobin values
Total hospitalization duration (in days)

Patient health at admission was assessed using the American Society of Anesthesiologists (ASA) risk classification [14] and the Charlson Comorbidity Index (CCI) [15].

Data was extracted from the hospital information system and laboratory database. Fracture type classification was based on radiographs taken at the time of admission and later retrieved from the hospital picture archiving and communication system (PACS) for analysis. The type of fracture was determined by two of the authors (AB and AO) through independent review of the PACS images. In case of disagreement, the senior author (AD) assessed the radiographs, and the fracture type was determined by consensus.

For patients who sustained a fracture while already hospitalized, the time of admission was defined as the time of fracture diagnosis. The time of discharge was defined as the moment the patient either exited the hospital or was transferred to another department within the hospital.

Descriptive statistical analysis was performed to summarize patient characteristics and clinical outcomes using GraphPad Prism 10 (Version 10.4.1, San Diego, CA, USA). Continuous variables are presented as means with standard deviations (SD) or as medians with interquartile ranges (IQR), as appropriate. Categorical variables are summarized as counts and percentages.

## Results

**Fig. 1:**
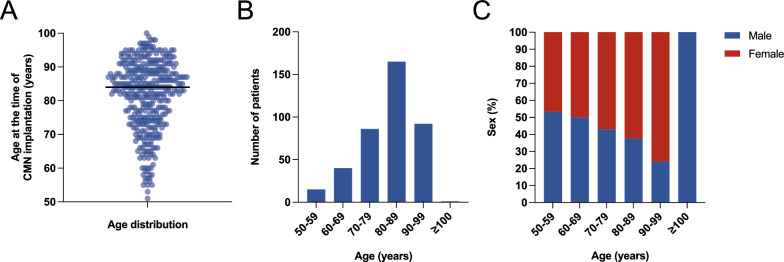
Age and sex distribution of the study population. **A** Age distribution of the study population, with a mean age of 84 years. **B** Age distribution is categorized into groups, illustrating the proportion of patients in each age range. **C** Sex distribution across age groups shows an increasing proportion of female patients with advancing age

The study population consisted of 401 patients, with a higher proportion of females (62.6%) compared to males (37.4%). The median age of patients was 84 years (IQR 75–89, Fig. 1A). The highest number of patients was seen in the 80–90 years group (Fig. 1B). Within the age groups, the percentage of females increased with an increase in age (Fig. 1C).

**Fig. 2:**
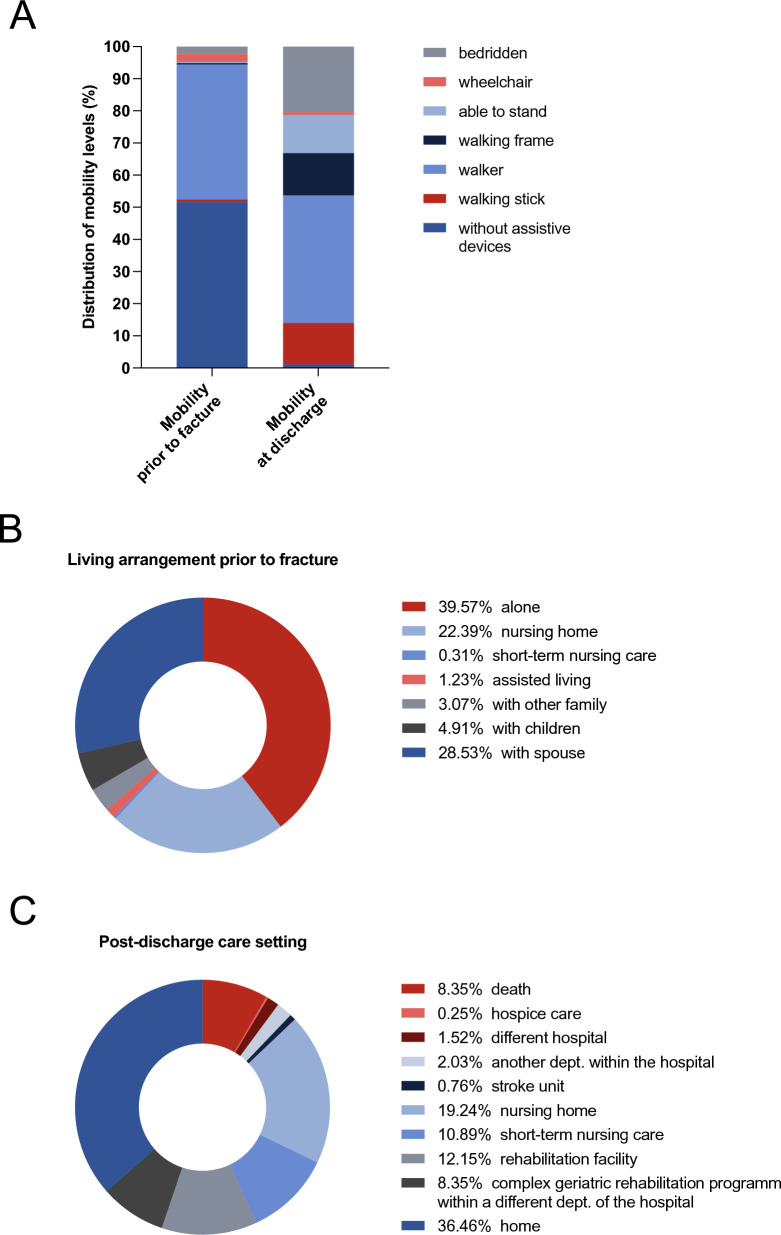
Mobility changes, living situation prior to fracture and post-discharge care setting. **A** Pre-fracture mobility status compared to mobility at discharge, illustrating the marked decline in functional independence. **B** Living arrangements before the fracture, showing the proportion of patients living alone, with family, or in nursing facilities. **C** Discharge destinations of patients following hospitalization

Prior to the fracture and hospital admission, 51.6% of patients were fully mobile without any walking aids. A small proportion of patients (2.4%) were bedridden, while another 2.4% used a wheelchair. Only 0.4% of patients were able to stand but could not walk, and another 0.4% required a walking frame. A large proportion (42.1%) relied on a walker as a walking aid, while 0.7% used a walking stick. At discharge, a marked decline in mobility was observed. Only 1% of patients remained able to walk without any walking aids. The percentage of bedridden patients increased to 20.5%, while 3% required a wheelchair. The proportion of patients who could stand but not walk increased to 11.9%, and 13.2% required a walking frame. The majority, 39.6%, used a walker, while 13% depended on a walking stick or crutches (Fig. 2A).

Before the fracture and hospital admission, 28.5% of patients lived with their spouse, 4.9% lived with their children, and 3.1% lived with other family members. A small proportion of patients, 1.2%, lived in assisted living facilities, while 0.3% were in short-term nursing care. Nursing homes accounted for 22.4% of living arrangements, while 39.6% of patients lived alone (Fig. 2B).

Regarding post-discharge care settings, 36.5% of patients returned home, while 8.4% were transferred to a complex geriatric rehabilitation program within a different department of the hospital. A total of 12.2% were discharged to a rehabilitation facility, 10.9% to short-term nursing care, and 19.2% to a nursing home. Additionally, 4.3% were transferred to another department of the hospital and 0.3% to hospice care. All-cause mortality during hospitalization was recorded in 8.4% of cases (Fig. 2C).

**Fig. 3:**
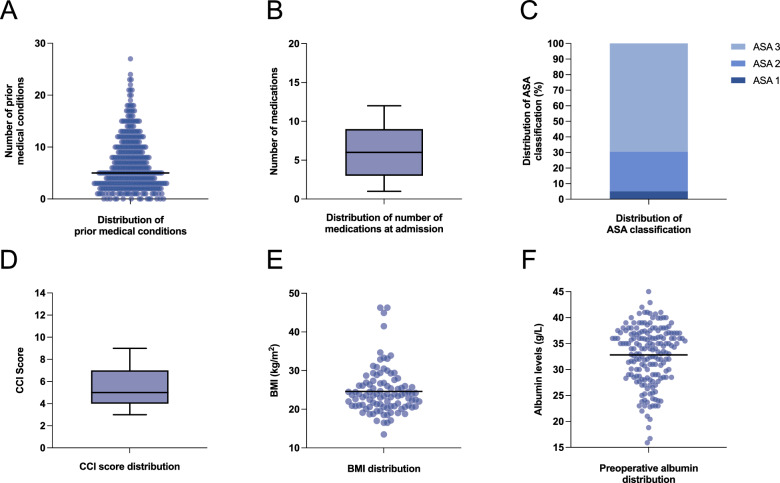
Pre-existing medical conditions, ASA score, BMI, and preoperative albumin values. **A** Number of pre-existing medical conditions at admission (median = 5). **B** Number of prescribed medications at admission (median = 6). **C** ASA classification of patients at the time of surgery, reflecting preoperative health status. **D** Charlson Comorbidity Index score distribution (median = 5). **E** Distribution of body mass index (BMI) at admission (mean = 24.6 kg/m^2^). **F** Preoperative albumin levels (mean = 32.8 g/L). Whiskers of box plots represent the 10th–90th percentile

The median number of pre-existing medical conditions at admission was 5 (IQR 3–10, Fig. 3A), while the median number of prescribed medications was 6 (IQR 3–9, Fig. 3B). The ASA classification distribution indicated that 5.1% of patients were categorized as ASA 1, 25.4% as ASA 2, and 69.5% as ASA 3 (Fig. 3C).

At admission, 26.1% of patients were receiving baseline osteoporosis therapy, while 3.5% were on specific osteoporosis therapy. Anticoagulants and platelet aggregation inhibitors were prescribed in 53% of cases. The median CCI score was 5 (IQR 4–7, Fig. 3D), and the mean BMI was 24.6 (Fig. 3E). Mean albumin levels at admission were 32.8 g/L (± 5.5 SD, Fig. 3F).

**Fig. 4:**
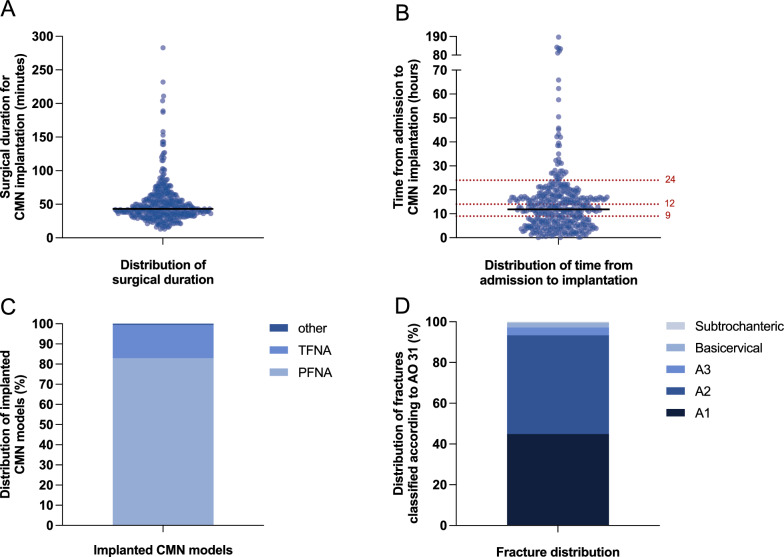
Parameters related to the surgical procedure. **A** Duration of surgery in minutes (median = 43 min). **B** Time from hospital admission to cephalomedullary nailing in hours (median = 11.9 h). Data points were stratified into four groups based on their frequency distribution: < 9 h, 9–12 h, 12–24 h, and > 24 h. **C** Distribution of implanted nail models among patients who underwent cephalomedullary nailing. **D** Fracture classification of the study population based on the AO/OTA system

The median duration of surgery was 43 min (IQR 34–57, Fig. 4A), while the median time from admission to implantation was 11.9 h (IQR 5.9–17.2, Fig. 4B). Regarding intraoperative parameters, the most implanted nail model was the Proximal Femoral Nail Antirotation (PFNA; Synthes; Oberdorf, Switzerland), used in 82.8% of cases, followed by the Trochanteric Femoral Nail Advanced (TFNA; DePuy Synthes; Zuchwil, Switzerland) in 16.7% of cases, with two patients receiving other implants (Fig. 4C). A closed reduction and osteosynthesis was performed in 92% of cases, whereas an open reduction was necessary in the remaining 8% due to unsuccessful closed reduction. Cement augmentation was carried out in 10.5% of cases, and cerclage wires were applied in 6.2%. General anesthesia was administered in 63.4% of cases, while 36.6% of patients underwent spinal anesthesia.

Fractures were predominantly located on the right side of the body (56.3% of the cases). Fracture classification based on the AO/OTA system revealed that 46.2% of fractures were type A1, 49.9% were type A2, and 3.9% were type A3 while 2.3% were basicervical fractures and 0.5% subtrochanteric fractures (Fig. 4D). Additional injuries requiring intervention were observed in 8.3% of patients. Within this group, distal radial fractures were the most commonly observed concomitant injuries (2.5%).

**Fig. 5:**
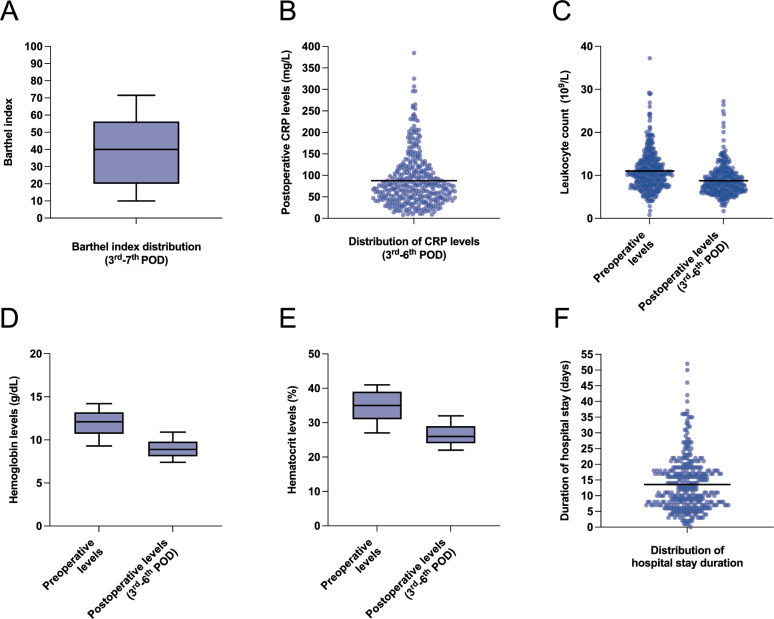
Postoperative outcomes. **A** Barthel index recorded between postoperative days 3 and 7, assessing functional independence (median = 40). **B** Postoperative C-reactive protein (CRP) levels measured between postoperative days 3 and 6 (mean = 87.7 mg/L). **C** Pre- and postoperative leukocyte count (mean of preoperative levels = 11.0 × 10^9^/L, mean of postoperative levels = 8.8 × 10^9^/L). **D** Pre- and postoperative hemoglobin levels (mean of preoperative levels = 11.9 g/dL, mean of postoperative levels = 9.1 g/dL). **E** Pre- and postoperative hematocrit levels (mean of preoperative levels = 34.7%, mean of postoperative levels = 26.6%). **F** Length of hospital stay in days (median = 12 days). Whiskers of box plots represent the 10th–90th percentile

Postoperative parameters revealed a median Barthel Index of 40 (IQR 20–56.3, Fig. 5A). A total of 30.2% of patients were enrolled in a complex geriatric rehabilitation program, while 36.7% required a postoperative blood transfusion.

Laboratory values between the 3rd and 6th POD showed a mean postoperative CRP level of 87.7 mg/L (± 60.7 SD, Fig. 5B). The mean leukocyte count was 11.0 × 10^9^/L preoperatively (± 4.6 SD) and decreased to 8.8 × 10^9^/L postoperatively (± 3.5 SD, Fig. 5C). The mean preoperative hemoglobin (Hb) level was 11.9 g/dL (± 2.0 SD), which decreased to 9.1 g/dL (± 1.4 SD) postoperatively (Fig. 5D). The mean hematocrit level was 34.7% preoperatively and dropped to 26.6% postoperatively (Fig. 5E). Revision surgery during the hospital stay was necessary in 4.5% of patients due to infection, mechanical complications, or peri-implant fractures. 12.3% of all patients required an ICU stay. The median length of hospital stay was 12 days (IQR 7–18, Fig. 5F).

## Discussion

Per-, sub-, and intertrochanteric femur fractures represent a critical challenge in elderly patients, often signifying a turning point toward reduced independence and increased healthcare needs [16]. This study analyzed 401 patients undergoing cephalomedullary nailing for these fractures, focusing on demographic characteristics, pre-existing medical conditions, and postoperative outcomes.

The median patient age of 84 years aligns with the established link between advanced age and osteoporotic fractures [17]. The increasing proportion of female patients with age further underscores the well-established role of osteoporosis in fracture risk. Postmenopausal estrogen deficiency leads to accelerated bone resorption [18, 19], rendering elderly women particularly susceptible to fragility fractures. This trend is exacerbated by the demographic shift toward an aging population in countries such as Germany [20], where increasing life expectancy contributes to a rising number of osteoporotic fractures and associated healthcare burdens.

The findings of this study reinforce that per-, sub- and intertrochanteric fractures should be regarded as prototypical osteoporosis-associated fractures. The high prevalence of these fractures in elderly women aligns with previous epidemiological data showing that postmenopausal bone loss, compounded by age-related reductions in bone density [21, 22], significantly increases the likelihood of low-energy fractures [23, 24]. Given that conservative management is rarely a viable option due to the rapid functional decline and high risk of complications such as deep vein thrombosis, pneumonia, and pressure ulcers [25, 26], surgical intervention remains the standard of care [27, 28]. However, while surgery provides mechanical stabilization, it does not fully mitigate the consequences of the fracture, particularly regarding long-term functional outcomes or reduced mobility [29, 30].

The decrease of patient mobility underscores the severe effect of these fractures on independence. Before injury, over half of patients were fully mobile without aids, showing that many elderly remain independent despite prevalent osteoporosis [31]. However, following hospitalization, mobility was markedly reduced. After hospitalization, patients experienced a marked decline in mobility, with many becoming bedridden or dependent on walking aids, and very few regaining their previous level of independence. While several studies have confirmed a significant decline after implantation of a CMN following a pertrochanteric femur fracture [32, 33], it is important to note that the presented data represent mobility status at discharge to post-discharge care settings mentioned above, including discharge to another department of the hospital. Thus, many patients may recover further functional independence over subsequent months.

Several factors likely contribute to this deterioration. Pain and postoperative muscle atrophy can severely limit the ability to regain pre-fracture mobility [34, 35]. Additionally, many elderly patients develop a psychological fear of falling [36], which discourages movement and leads to further functional decline. The high prevalence of pre-existing comorbidities, as reflected by the median of five medical conditions per patient, further complicates postoperative recovery. Diabetes, cardiovascular disease, and cognitive impairment can delay rehabilitation, while polypharmacy introduces risks of drug interactions [37, 38]. This can contribute to postural instability and delay recovery [39].

The observed postoperative decline in hemoglobin, accompanied by a reduction in hematocrit, likely reflects a combination of intraoperative blood loss and hemodilution due to intravenous fluid administration [40]. This can exacerbate postoperative fatigue, prolong recovery, and contribute to increased cardiovascular strain [41, 42]. Additionally, recent research suggests that additional hematological parameters, such as the neutrophil-to-lymphocyte ratio, can serve as predictors of postoperative mortality, reflecting the interplay between inflammatory response and physiological stress in elderly hip fracture patients [43].

While a substantial number of patients were receiving baseline osteoporosis therapy at admission (26.1%), only a small proportion were on specific osteoporosis treatment, suggesting that many cases of osteoporosis remain either undiagnosed or inadequately managed. Similarly, the documentation of BMI reveals a potential bias. The recorded BMI values indicate a relatively high mean of 24.6, suggesting that overweight or obesity was prevalent in the documented cases. However, BMI was frequently not recorded (recorded in 24.4% of cases), and when it was, it tended to be in patients who were obese. This selective documentation likely leads to an overestimation of the average BMI in this cohort, while underweight or malnourished individuals, who are at an elevated risk for poor postoperative outcomes, may be underrepresented in the available data.

## Limitations

This study has several limitations. The retrospective design increases the risk of documentation bias and incomplete data, notably for BMI and comorbidities. Additionally, DXA values could not be included in this study. The WHO definition of osteoporosis is a T-score ≤ − 2.5 SD. Nevertheless, presence of a fragility fracture alone establishes a clinical diagnosis of osteoporosis, irrespective of bone mineral density (BMD) measurements [44]. Hence, osteoporosis was present in our patient collective.

Additional limitations include the absence of long-term follow-up of sustained functional outcomes and late complications. The single center setting limits generalizability; thus, future prospective and multicenter studies are required.

## Conclusions

This study highlights the significant impact of per-, sub-, and intertrochanteric fractures on elderly patients, emphasizing the pronounced decline in mobility and independence following these injuries. Our findings demonstrate a substantial reduction in postoperative mobility, accompanied by increased dependence on assistive walking devices and a notable decrease in hemoglobin and hematocrit levels, reflecting considerable physiological stress and increased morbidity. Additionally, underreporting of medical conditions, particularly osteoporosis, and malnutrition, combined with biases in BMI documentation, likely contributes to suboptimal patient management. Therefore, a comprehensive multidisciplinary approach, including proactive osteoporosis screening and management, as well as careful perioperative care, and structured early rehabilitation is critical to reduce complications, preserve patient autonomy, and enhance overall recovery outcomes in this vulnerable population.

## Data Availability

The datasets used and/or analyzed during the current study are available from the corresponding author on reasonable request.

## Abbreviations

CMN: Cephalomedullary Nails
PACS: Picture Archiving and Communication System
SD: Standard deviation
IQR: Interquartile range
ASA: American Society of Anesthesiologists
CCI: Charlson Comorbidity Index
BMI: Body Mass Index
PFNA: Proximal Femoral Nail Antirotation
TFNA: Trochanteric Femoral Nail Advanced
ICU: Intensive care unit
POD: Postoperative day
CRP: C-reactive protein
DXA: Dual-energy X-ray Absorptiometry
WHO: World Health Organization
BMD: Bone mineral density
